# Low-intensity body building exercise induced rhabdomyolysis: a case report

**DOI:** 10.1186/1757-1626-2-7

**Published:** 2009-01-05

**Authors:** Massimiliano Gagliano, Daniela Corona, Giuseppe Giuffrida, Alessia Giaquinta, Tiziano Tallarita, Domenico Zerbo, Massimiliano Sorbello, Annalaura Paratore, Carla Virgilio, Alessandro Cappellani, Pierfrancesco Veroux, Massimiliano Veroux

**Affiliations:** 1 Department of Surgery, Transplantation and Advanced Technologies; Vascular Surgery and Organ Transplant Unit, University Hospital of Catania, Via Santa Sofia, 86 95123 Catania, Italy; 2 Department of Surgery, Transplantation and Advanced Technologies; Anaesthesia and Intensive Care Unit, University Hospital of Catania, Via Santa Sofia, 86 95123 Catania, Italy

## Abstract

**Introduction:**

Rhabdomyolysis is a severe and debilitating condition that promotes muscle breakdown and is a relatively rare, not always diagnosed cause of acute renal failure (ARF) with an 8–20% reported incidence. Exertional rhabdomyolysis only appears in adult patients 24–48 h after strenuous activities as military basic training, weight lifting, and marathon running.

**Case presentation:**

A 30-year-old man was admitted to our department because of weakness and painful swelling of the muscles as well as dark urine appearing 24 h after carrying out a body-building exercises of low intensity. The development of an acute exertional rhabdomyolysis was confirmed by the increased serum enzyme levels and myoglobinuria. The patient was treated with intravenous sodium chloride, and sodium bicarbonate. The nephrotoxicity of myoglobin was decreased by forced alkaline diuresis.

**Conclusion:**

The reported case emphasizes the occurrence of acute rhabdomyolysis even in those who underwent a low-intensity exercise. A proper treatment is mandatory to avoid a sudden worsening of clinical conditions eventually evolving to acute renal failure.

## Introduction

Rhabdomyolysis is a severe and debilitating condition that promotes muscle breakdown and is a relatively rare, not always diagnosed cause of acute renal failure (ARF) with an 8–20% reported incidence [1,2]. Exertional rhabdomyolysis only appears in adult patients 24–48 h after strenuous activities as military basic training, weight lifting, and marathon running [3]. Strenuous exercise can result in damage to skeletal muscle cells and in most cases is resolved without consequences. However, in case of severe damage, there is a huge release of myoglobin which, in high concentrations and under certain conditions, such as dehydration and heat stress, may precipitate in the kidneys, thereby resulting in ARF [1,2,4]. The classical clinical presentation of rhabdomyolysis is characterized by myalgias, muscle weakness, and darkened urine [4]. Laboratory results may include delayed high levels of muscle enzymes in the plasma (CPK, ALT, AST, LDH) myoglobinemia and myoglobinuria, disturbance in blood electrolyte balance, and in severe cases, disturbance in coagulatory function and DIC [2,4]. The main treatment for rhabdomyolysis is fluids administration and maintaining urination, in order to preserve renal function [4]. The treatment results and mortality rate were primarily influenced by the patients' general condition at admission. A favourable outcome may be obtained especially in those patients who were hospitalized in a nephrological center[2].

We report a unique case of acute rabdomyolysis induced by a low-intensity body building exercise.

## Case presentation

A 30-year-old man was admitted to our department because of weakness and painful swelling of the muscles as well as dark urine appearing 24 hours after carrying out a body-building exercise of low intensity. The development of an "acute exertional rhabdomyolysis" was confirmed by the increased serum enzyme levels and myoglobinuria. Total serum creatine phosphokinase (CPK) was 70,962 IU/L (normal range (n.r) 35 – 175 IU/L). The laboratory tests revealed a serum creatinine of 2.1 mg/dl and the serum LDH level was 4,981 IU/L (n.r 250 – 500 IU/L) with an elevation of the serum ALT and AST levels of 347 IU/l (n.r 5 – 40 IU/L) and 1,205 IU/L (n.r 5 – 42 IU/L), respectively. A myoglobin serum concentration of 1,702 ng/ml (n.r 28 – 72 ng/mL) was also found. A diagnosis of pigmenturia due to urinary myoglobin excretion was suspected, and the patients was immediately treated with 3000 ml of intravenous sodium chloride, and 500 ml of sodium bicarbonate. The nephrotoxicity of myoglobin was decreased by forced alkaline diuresis. The patient did well after two days, and CPK, AST and ALT values gradually restored after ten days.

## Discussion

Cases of rhabdomyolysis associated with other forms of excessive physical exertion are commonly reported, however cases associated with low-intensity and weight lifting are very rarely reported, probably because most cases in this setting go unrecognized and are diagnosed as simple muscle strain [5]. The exact incidence of rhabdomyolysis in this setting of body building exercise remains unknown. The reported case emphasizes the occurrence of acute rhabdomyolysis even in those who underwent a low-intensity exercise. A proper treatment is mandatory to avoid a sudden worsening of clinical conditions eventually evolving to acute renal failure.

## Abbreviations

AST: Aspartate Aminotransferase; ALT: Alanine AminoTransferase; CPK: Creatine Posphokinase; DIC: Disseminated Intravascular Coagulation; LDH: Lactic dehydrogenase

## Consent

Written informed consent was obtained from the patient for publication of this case report. A copy of the written consent is available for review by the Editor-in-Chief of this journal.

## Competing interests

The authors declare that they have no competing interests.

## Authors' contributions

MG was a major contributor in writing the manuscript. DC interpreted the laboratory data. GG has been involved in the draft of the manuscript. AG interpreted the data regarding the follow up of the patient. TT interpreted the data regarding the follow up of the patient. DZ was responsible of the management of the patient. MS was responsible of intensive care management. AP was responsible of intensive care management. CV has been involved in the draft of the manuscript. AC has been involved in the draft of the manuscript. PV was responsible of the management of the patient. MV was a major contributor in writing the manuscript and has given the final approval to the manuscript.
